# Impact of prenatal stress on mother-infant dyadic behavior during the still-face paradigm

**DOI:** 10.1186/s40479-018-0078-8

**Published:** 2018-01-22

**Authors:** Isabell Ann-Cathrin Wolf, Maria Gilles, Verena Peus, Barbara Scharnholz, Julia Seibert, Christine Jennen-Steinmetz, Bertram Krumm, Marcella Rietschel, Michael Deuschle, Manfred Laucht

**Affiliations:** 1 Department of Psychiatry and Psychotherapy, Central Institute of Mental Health, Medical Faculty Mannheim / Heidelberg University, J 5, 68159 Mannheim, Germany; 2 Clinic for General Psychiatry, Center for Psychosocial Medicine, Heidelberg University Hospital, University of Heidelberg, Heidelberg, Germany; 3 Department of Biostatistics, Central Institute of Mental Health, Medical Faculty Mannheim, University of Heidelberg, Mannheim, Germany; 4 Department of Genetic Epidemiology in Psychiatry, Central Institute of Mental Health, Medical Faculty Mannheim, University of Heidelberg, Mannheim, Germany; 5 Department of Child and Adolescent Psychiatry and Psychotherapy, Central Institute of Mental Health, Medical Faculty Mannheim, University of Heidelberg, Mannheim, Germany; 6 Department of Psychology, University of Potsdam, Potsdam, Germany

**Keywords:** Prenatal stress, Face-to-face still-face paradigm, Resilience, Psychosocial stress, Cortisol

## Abstract

**Background:**

Mother-infant interaction provides important training for the infant’s ability to cope with stress and the development of resilience. Prenatal stress (PS) and its impact on the offspring’s development have long been a focus of stress research, with studies highlighting both harmful and beneficial effects. The aim of the current study was to examine the possible influence of both psychological stress and hypothalamic-pituitary-adrenal (HPA) axis activity during pregnancy with mother-child dyadic behavior following stress exposure.

**Methods:**

The behavior of 164 mother-infant dyads during the still-face situation was filmed at six months postpartum and coded into three dyadic patterns: 1) both positive, 2) infant protesting-mother positive, and 3) infant protesting-mother negative. PS exposure was assessed prenatally according to psychological measures (i.e., psychopathological, perceived and psychosocial PS; *n* = 164) and HPA axis activity measures (maternal salivary cortisol, i.e., cortisol decline and area under the curve with respect to ground (AUCg); *n* = 134).

**Results:**

Mother-infant dyads in both the high- and low-stress groups showed decreasing positive and increasing negative dyadic behavior in the reunion episode, which is associated with the well-known “still-face” and “carry-over” effect. Furthermore, mother-infant dyads with higher psychosocial PS exhibited significantly more positive dyadic behavior than the low psychosocial PS group in the first play episode, but not in the reunion episode. Similarly, mother-infant dyads with high HPA axis activity (i.e. high AUCg) but steeper diurnal cortisol decline (i.e. cortisol decline) displayed significantly less negative behavior in the reunion episode than dyads with low HPA axis activity. No significant results were found for psychopathological stress and perceived stress.

**Conclusions:**

The results suggest a beneficial effect of higher psychosocial PS and higher prenatal maternal HPA axis activity in late gestation, which is in line with “stress inoculation” theories.

**Electronic supplementary material:**

The online version of this article (10.1186/s40479-018-0078-8) contains supplementary material, which is available to authorized users.

## Background

Early mother-infant interaction plays a pivotal role in the infant’s development of emotion regulation, which is essential for the development of resilience [1, 2]. In the mutual interaction with their caregivers, infants learn and train age-appropriate self-regulation strategies when confronted with everyday stressors. This allows for the creation and integration of new experiences, enabling infants to accomplish age-related developmental tasks [3, 4]. Previous studies have highlighted the role of mother-infant dyadic behavior not only in the children’s vocalization [5], but also in the brain development in the first year of life [6]. Moreover, mother-infant attachment has been identified as a beneficial factor in the cognitive development of prenatally stressed infants [7]. For example, Conway and McDonough [8] reported an association between maternal sensitivity during infancy and the children’s resilience during preschool age. In their review, Leclère and colleagues [9] emphasized the crucial role of synchrony in mother-infant behavior in terms of contributing to benefits or vulnerabilities in the infant. The majority of recent studies focusing on early life stress (ELS) and its role in the development of health and disease, as well as resilience [10–13], suggest that ELS, and especially prenatal stress (PS), has an important impact on epigenetic alterations in the DNA and thus on changes in the hypothalamic-pituitary-adrenal (HPA) axis [14]. “Stress sensitization” and “stress inoculation” theories represent conflicting positions concerning the impact of PS on adolescent or adult life [15–18].

According to the “*stress sensitization model*”, exposure to PS can subsequently lead to negative consequences later in life, such as higher prevalences of psychiatric disorders, e.g. anxiety disorders, depression, attention-deficit/hyperactivity disorder or autism spectrum disorders [11, 19–21]. The underlying process is known as “fetal programming”, defined by Glover and colleagues [22] as the alteration of infants’ early development due to changes in the direct environment (i.e. in utero). Studies have found that in normal pregnancy, 10–20% of maternal cortisol crosses the placental barrier [23]. Therefore, maternal cortisol can have a major effect on fetal cortisol concentrations, and is able to double them. However, when the mother-to-be experiences more stress, a down-regulation of maternal 11ß-hydroxysteroid dehydrogenase 2 (11ß-HSD2) due to complications, maternal stress, and adversities might lead to a reduced protective enzymatic effect and a further elevation of the maternal glucocorticoids passing the placental barrier [24–27]. Accordingly, infants who are overexposed to glucocorticoids may suffer from long-term alterations, mainly referred to as epigenetic methylation of the DNA [13]. The severity of these alterations is influenced by gene-environment interactions, which depend on several factors such as the timing (i.e., sensitive time frames), duration, and quantity of stressors [22].

In contrast, according to the “*stress inoculation model*”, increased prenatal stress can be beneficial in terms of increasing hardiness and resilience [28–30]. This theory posits that infants exposed to ELS experience a so-called “steeling effect” [31], resulting in less reactivity to similar future stressors [17]. In their “*match/(mis-)match hypothesis*”, Nederhof and Schmidt [32] combined stress sensitization and stress inoculation theories. They assumed that a match of the early (prenatal) environment with the later adult (postnatal) environment would lead to a better adaptation and thus to a benefit in the offspring, while a mismatch would lead to an elevated disease vulnerability later in life [33].

Regarding the interaction with caregivers in the first years of life, Tronick and Beeghly [3] suggested in their “*mutual regulation model”* that the development of the infant’s emotion regulation relied on the constant training of matching dyadic mother-infant behavior and the reparation of mismatching dyadic behavior states. The still-face paradigm is a well-known experimental method to examine the infant’s management of an acute stressor. It explores the infant’s capacity to cope with induced stress during a mother-infant play situation [34, 35]. Infants’ reactions to the still-face paradigm have been shown to be stable over short time intervals [36], with numerous studies reporting a typical “still-face effect”, characterized by a decrease in infant positive behavior and an increase in infant protesting behavior, as well as an increase in self-regulating behavior (i.e.; touching the mouth, thumb-sucking, hand-to-mouth movements) following the stressful still-face episode [37–39].

Conway and McDonough [8] employed the still-face paradigm during mother-infant interaction, and found that maternal sensitivity, but not infants’ negative affect, predicted resilience in preschool children. Further, Müller and colleagues [40] reported an association between the latency of mismatching states in the mother-infant dyad during the still-face paradigm and the infants’ salivary cortisol responses. Along with further studies on mother-infant synchrony, research findings on the impairing influence of disturbed mother-infant dyads on child development [41–43] underlined the important role of “contingent reciprocity” in mother-child interaction [44]. For example, mother-infant dyads with depressive mothers, demonstrated less maternal positivity and increased negative affect, and infants showed increased negative, depressive-like affect compared to controls [45–47]. Interestingly, a study in mothers with borderline personality disorder (BPD) found that their three-month-old infants had generally less positive vocalization and showed less nonautonomic self-regulation during the still-face paradigm compared to controls [48]. Moreover, the infants seemed especially troubled by the still-face episode resulting in decreased infant gazing behavior. The mothers with BPD seemed to be more challenged during the reunion episode after the stressor when resuming the play, and showed less smiling and more intrusive behavior [48].

Concerning maternal HPA axis activity, prenatal maternal morning cortisol was found to be associated with children’s HPA axis reactions to the first day in school after the summer break [49]. Previous research also revealed prenatal maternal cortisol to be positively associated with early negative infant affect and behavior, resulting in more infant crying and fussing at age five months [50]. On the other hand, it may be not only that maternal HPA axis activity relates to future infant behavior, but also that maternal behavior is associated with future HPA axis activity in the offspring. Schmid et al. [51] demonstrated that less maternal stimulation during early mother-infant interaction predicted later diminished plasma adrenocorticotropic hormone (ACTH) and cortisol increase in 19-year-old male offspring experiencing acute psychosocial stress. In view of the essential role of the HPA axis in coping with stress, early PS experiences and related alterations in HPA axis function have been discussed to lead to prolonged reactions to stressors, which could be related to infant behavior and temperament as well as later disease propensity (e.g., depression; [52]).

**Fig. 1:**
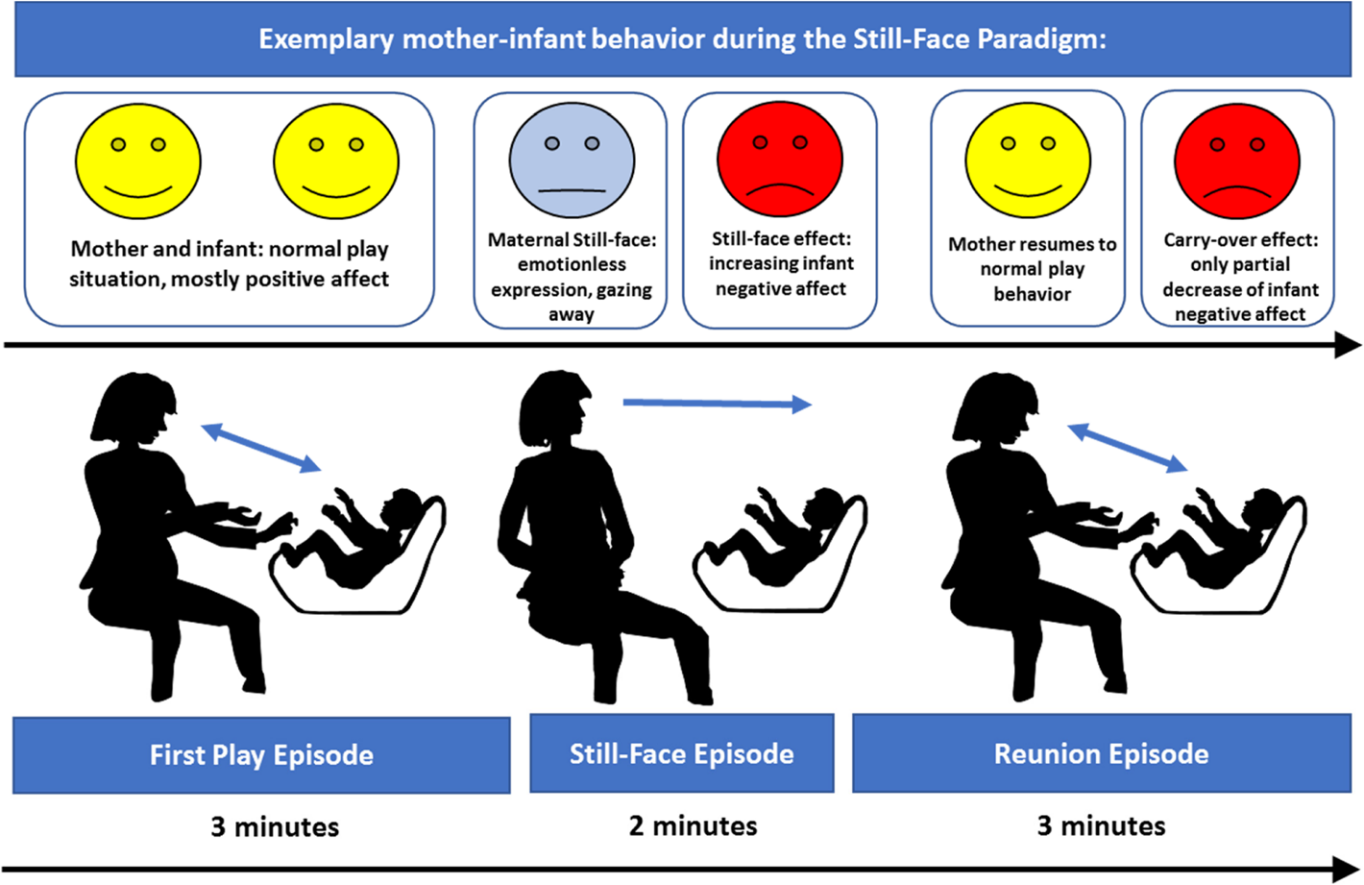
Exemplary Still-Face Paradigm procedure with 3′-2′-3′ time intervals revealing the still-face and carry-over effect

Taken together, these mixed results generated a background for further research on the impact of PS on mothers and infants. To our knowledge, the present study is the first to examine the potential influence of HPA axis and psychological stress in pregnancy with mother-infant dyadic behavior in the still-face paradigm, while reacting to an acute induced stressor (i.e.; still-face procedure). Given that previous studies provided evidence for both a beneficial and an adverse impact of prenatal stress on mother-infant dyadic behavior [17, 53], we tested for both potential outcomes. Furthermore, we expected less positive infant behavior in the still-face episode and more negative infant affect provoked by the “still-face effect”. Based on previous research using the still-face paradigm, we expected an overall increase in negative infant behavior after the still-face episode, seen as a “carry-over effect” of the “still-face effect” (see Fig. 1 and [39]).

## Methods

### Participants

Expectant mothers were participating in the “Pre-, Peri- and POstnatal Stress: Epigenetic Impact on DepressiON” (POSEIDON) study and were recruited in their third trimester of pregnancy (*N* = 410, 4–8 weeks prior to term) in three obstetric clinics in the Rhine-Neckar- region of Germany (see Dukal et al. [54] for further information). The study protocol was approved by the Ethics Committee of the Medical Faculty Mannheim of the University of Heidelberg and the Ethics Committee of the Medical Association of Rhineland-Palatinate, and was conducted in accordance with the Declaration of Helsinki. All mothers provided written informed consent prior to enrolment in the study. Participation in the still-face paradigm six months after birth was voluntary. Inclusion criteria for the mothers-to-be were: German-speaking; main caregiver; and age 16–45 years. Exclusion criteria were: hepatitis B or C, human immunodeficiency virus (HIV) infection; any current psychiatric disorder requiring inpatient treatment; any history of current diagnosis of schizophrenia/psychotic disorder; or any substance dependency other than nicotine during pregnancy. The exclusion criteria for infants were birth weight < 1500 g; gestational age < 32 weeks; or the presence of any congenital diseases, malformations, deformations, and/or chromosomal abnormalities.

### Mother-infant behavior

Two-hundred mother-infant videos were collected based on an a-priori participant selection procedure that relied on a composite stress measure (i.e., total adversity score), which enabled the identification of the 100 most stressed and the 100 least stressed mothers (for details see Dukal et al. [54]). Several video-sets had to be excluded due to technical problems of the filmed material; for detailed information, see Additional file 1. For the analysis of maternal diurnal cortisol, data of 134 mother-infant dyads were available, as 30 dyads were excluded due to missing data (i.e., too little saliva provided, no return of samples; *n* = 17), outliers (≥ / ≤ 2 SD; *n* = 10), or implausible, impossible morning cortisol (FI and/or FII ≤ 7 nmol/l values; *n* = 3) (for details, see Wolf et al. [55]). We used a strict limit of ≥ / ≤ 2 SD to be able to filter the lowest outliers in morning cortisol scores (c.f. [56]). Statistical analyses examining the outliers for selection effects (e.g., gender, total adversity score, maternal age) were insignificant (all p’s > .05). For detailed maternal and infant characteristics, see Table 1 as well as [55].

**Table 1:** Means and standard deviations of behavior dyads (psychological stress groups and HPA axis activity groups)

	IposMpos dyad		IproMpos dyad		IproMneg dyad	
Psychological PS (*n* = 164)	FFE	RE	FFE	RE	FFE	RE
	M (SD)	M (SD)	M (SD)	M (SD)	M (SD)	M (SD)
Psychopathological PS	H: 6.13 (4.53)	H: 3.85 (3.76)	H: 1.30 (3.14)	H: 6.54 (10.16)	H: 0.43 (1.64)	H: 2.30 (4.78)
	L: 4.13 (4.68)	L: 3.82 (4.30)	L: 1.92 (4.27)	L: 5.97 (8.86)	L: 0.39 (1.32)	L: 1.74 (3.44)
Perceived PS	H: 6.42 (4.58)	H: 4.10 (3.64)	H: 1.01 (2.68)	H: 6.03 (9.10)	H: 0.42 (1.64)	H: 2.40 (4.81)
	L: 4.50 (4.57)	L: 3.58 (4.38)	L: 2.21 (4.51)	L: 6.49 (9.99)	L: 0.40 (1.36)	L: 1.64 (3.40)
Psychosocial PS	H: 6.76 (4.60)	H: 3.96 (3.81)	H: 0.93 (2.47)	H: 6.08 (10.16)	H: 0.36 (1.51)	H: 2.44 (5.05)
	L: 4.15 (4.32)	L: 3.72 (4.25)	L: 2.29 (4.60)	L: 6.43 (8.87)	L: 0.47 (1.47)	L: 1.60 (3.01)
HPA axis activity (*n* = 134)						
Cortisol decline	F: 5.77 (4.38)	F: 3.76 (3.89)	F: 1.54 (3.82)	F: 6.59 (9.23)	F: 0.58 (1.91)	F: 2.54 (3.99)
	S: 5.13 (4.67)	S: 3.90 (4.19)	S: 1.56 (3.63)	S: 5.49 (8.86)	S: 0.37 (1.30)	S:1.29 (3.18)
AUCg	H: 6.03 (5.28)	H: 4.31 (4.31)	H: 1.59 (3.69)	H: 5.62 (9.32)	H: 0.42 (1.37)	H: 1.37 (2.99)
	L: 5.02 (3.64)	L: 3.54 (3.93)	L: 1.58 (3.83)	L: 6.24 (8.59)	L: 0.55 (1.89)	L: 2.51 (4.21)

Abbreviations: *IposMpos*: Infant positive-mother positive; *IproMpos*: Infant protesting-mother positive; *IproMneg*: Infant protesting-mother negative, *FFE*: face-to-face/ play episode; *RE*: Reunion episode; *M*: mean, *SD*: standard deviation, *F*: flat decline, *S*: steep decline, *AUCg*: area under the curve with respect to ground

Videos were filmed at six months postpartum at the Central Institute of Mental Health, Mannheim or in the mothers’ homes. Mother-infant dyads performed the well-established still-face paradigm [37]. The paradigm consists of three episodes: 1) the first play episode (three minutes), in which the mother interacts normally with the child, 2) the still-face episode (two minutes), in which the mother stops the play and, remains silently sitting with an expressionless face in front of the child, without reacting to or looking at the child, and 3) the reunion episode (three minutes), in which the mother resumes the normal play (see Fig. 1). Mother-infant dyads were left alone during the episodes; toys and pacifiers could not be used. The start and the end of the episodes were indicated by a sound signal. Videos were filmed with two video cameras (Sony™ HDR-CX130), one focusing on the mother’s face and, the other focusing on the infant. The infants sat opposite to their mothers at the same level in a Maxi-Cosi™ or similar baby chair and were belted during the experiment.

For the coding procedure, the two videos were synchronized and transformed into one split-half screen video using Corel™ Videostudio Pro X4 software. Behavioral coding of the videos was conducted using Interact™ software (Mangold International GmbH 2013, Ver. 9.7.8) by a trained and certified Infant and Caregiver Engagement Phases (ICEP; [34, 57]] coder, who was blind to the mothers’ stress exposure. According to the ICEP coding system, all caregiver and infant behaviors were coded (for further details, see Additional file 1: Table A1). For data reduction, three dyadic mother-infant behavior categories were formed: 1) Infant positive-mother positive dyad (IposMpos) was coded when mothers showed social monitor/positive vocalization or social positive engagement and infants showed social positive engagement simultaneously; 2) Infant protesting-mother positive dyad (IproMpos) was coded when mothers showed social monitor/positive vocalization or social positive engagement and infants showed negative/protesting behavior (i.e., crying, distress, being fussy); 3) Infant protesting-mother negative dyad (IproMneg) was coded when mothers showed intrusive, social monitor/neutral vocalization or non-infant-focused engagement, with the infant showing protesting behavior. Calculations were performed using Interact™ software by summing up the time for which both partners showed the respective dyadic behavior at the same time during play. The codings were computed separately for each episode into percentages referring to the duration of the play episode.

### Assessment of stress

#### Subjective stress experience indices

Mothers were interviewed and given questionnaires during the final trimester of pregnancy (for further details, see Dukal et al. [54]). To provide different psychological stress measurements, we used three composite scores computed by principle component analysis distinguishing psychopathological, perceived, and psychosocial stress of the mother during pregnancy (for further information, see Additional file 1).

#### HPA axis activity

Salivary cortisol measures were acquired as a reliable indicator of total free plasma cortisol [58]. Maternal diurnal cortisol data were obtained via saliva samples using Salivettes (Sarstedt™, Leicester, UK), which contained an untreated cotton swab. Saliva samples were collected in the late third trimester of pregnancy during one “normal working day”. We chose a threefold determination based on the protocol of Lederbogen and colleagues [59]. Mothers were instructed to chew on the cotton swab immediately after awakening (FI), but while still in bed; 30 min after getting up (FII); and 14 h after awakening (FIII). Instructions included precaution information regarding meals, drinks, brushing one’s teeth and smoking. Mothers indicated the date and times of saliva collection and sent the probes back to the study coordinators. All samples were stored at −25 °C. After thawing, the samples were centrifuged for five minutes at 3000 rev/min, resulting in a clear supernatant of low viscosity. Salivary cortisol was measured by means of a time resolved immunoassay with fluorescence detection. The lower limit of detection was 0.43 nmol/l, with interassay and intraassay coefficients of variation of less than 10% across the expected range of cortisol levels. The mean week of gestation for the saliva collection was 36.77 (SD 1.89). The measure diurnal cortisol decline was computed as the difference between the evening cortisol score and the highest morning score (FI or FII – FIII), as the cortisol morning peak is expected 0–0.5 h after awakening [60]. The cortisol measure area under the curve with respect to ground (AUCg) was computed according to the formula by Pruessner and colleagues [61]. The AUCg indicates the total amount of cortisol concentration per day and is defined by a trapezoid formula, calculating the area under the diurnal cortisol decline.

#### Statistical analysis

All statistical analyses were performed using PASW Statistics 21 (SPSS Inc., Chicago, USA). To examine the relationships between the three dependent variables (i.e., types of dyadic behavior), Pearson’s r correlations between the mother-infant dyadic behavior categories, as well as between the psychological and HPA axis activity stress groups were computed. Furthermore, paired *t*-tests for the ICEP infant behavior codes “infant social positive engagement” and “infant negative/protesting behavior” were calculated in order to compare each play phase with one another. For each psychological stress index (i.e., maternal psychopathology, perceived stress, and psychosocial stress) and for each HPA axis parameter (i.e., prenatal maternal cortisol decline, and cortisol area under the curve with respect to ground (AUCg)), the corresponding stress variable was dichotomized via median splits to form two groups with high and low stress levels (see Additional file 1 for more details). To examine whether the stress groups (i.e., mothers with low and high stress) differed from each other in the still-face paradigm, we ran a series of repeated-measures analyses of covariance (ANCOVA), with group as the between-subjects factor, the still-face episodes (e.g.; play episode and reunion episode) as within-subjects factor and the covariates maternal age, infant gender, parity, and video setting (home vs. lab). As a second additional validation, we adjusted for further confounders and included the covariates breastfeeding, current maternal depression during pregnancy, Apgar score after five minutes, perinatal complications, and perceived stress six months postpartum (assessed via the Perceived Stress Scale, PSS [62]) were included. Significant interaction effects were followed up by post- hoc contrasts comparing the two stress groups separately for each episode. Furthermore, mediation analyses were computed to test for the possibility of maternal behavior mediating the relationship between prenatal stress and infant behavior, using regression analysis and bias-corrected bootstrapping with the PROCESS model tool [63]. We ran mediation analyses with z-standardized maternal behavior (i.e., positive and negative behavior) as a mediator between PS (i.e., psychosocial PS, cortisol decline) and z-standardized infant behavior (i.e., infant positive and protesting behavior) in the reunion episode, including the covariates gender, maternal age, parity, and video setting (home vs. lab) in a first step and the additional covariates current maternal depression during pregnancy, breastfeeding, Apgar score five minutes after birth, perinatal complications, and perceived stress six months postpartum (assessed via the PSS) in a second step.

## Results

Correlations between mother-infant dyadic behavior categories across the play episodes were significant (all p’s between < .001 and *p* = .014), with the exception of IposMpos in the first play episode and IproMneg in the reunion episode (*r* = − 0.003; *p* = .968; for details see Additional file 1: Table A2). Given the highly significant intercorrelations between the psychological stress variables (see Additional file 1: Table A3; *r* = 0.604 to 0.739; all p’s < .001), we decided to assess the impact of the distinct stress dimensions separately in order to examine specific effects, similar to previous findings from our group by Dukal [54] and Nieratschker [64]. HPA axis activity and psychological stress measures showed a significant negative association of cortisol decline with psychopathological stress (*r* = −0.203; *p* = .019) and psychosocial stress (*r* = −0.184; *p* = .033), whereas perceived stress was unrelated to cortisol decline (*r* = −0.003; *p* = .974). Moreover, the cortisol AUCg was unrelated to the three psychological stress measures (r between - 0.061 and 0.081; all p’s > .360; see Additional file 1: Table A4).

Cortisol AUCg and cortisol decline were significantly positively correlated (*r* = 0.398; *p* < .001). Moreover, as expected, paired *t*-tests for the infant behavior showed significant episode effects between the first play and the still-face episode (*t* (163) = 14.64; p < .001), indicating a decrease in positive behavior, both for the still-face episode and the reunion for infant positive behavior (*t* (162) = −12.51; *p* < .001) and an increase in positive behavior. Furthermore, the paired *t*-test for infant positive behavior showed a significant decrease in positive behavior between the first play and reunion episode (*t* (162) = 3.04; *p* = .003). The results additionally revealed a significant episode effect on infant protesting behavior between the first play episode and the still-face episode (*t* (163) = −6.64; *p* < .001), with an increase in protesting behavior, but not for the still-face episode and the reunion episode (*t* (162) = −1.83; *p* = .070). However, a paired *t*-test for infant protesting behavior between the first play episode and the reunion episode showed a significant increase in negative behavior (*t* (162) = −8.28; *p* < .001).

### Impact of subjective psychological PS on mother-infant dyadic behavior during the still-face paradigm

#### Psychosocial PS

**Fig. 2:**
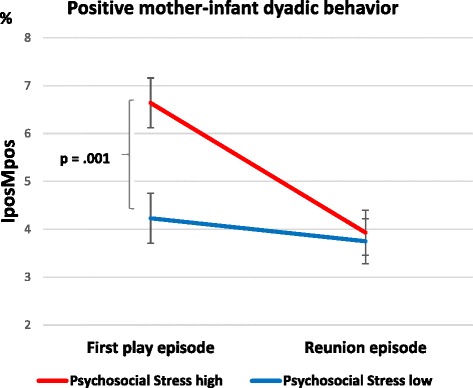
Positive mother-infant dyadic behavior depending on psychosocial PS groups during the play and reunion episode (Means and standard errors adjusted for covariates and significant contrasts)

The psychosocial PS x episode interaction showed a significant effect with regard to positive dyadic behavior (F(1,155) = 9.060, p = .003, partial η^2^ = .055), indicating that the effect of stress group differed depending on the play episode (for details, see Table 2). Post-hoc contrasts revealed that, in the first play episode, the low-psychosocial PS group showed more positive dyadic behavior (*p* = .001) than the high-psychosocial PS group, while this was not the case in the reunion episode (*p* = .793; see Fig. 2).

**Table 2:** Effect of psychosocial PS on mother-infant positive dyadic behavior. Results of ANCOVA^a^

Effect	IposMpos dyad		
	F/ (df)	*p*	Part. Eta Sq.
Psychosocial PS	4.721 (156)	.031	.029
Episode	0.140 (156)	.709	.001
Psychosocial PS x episode IA	9.647 (156)	.002	.058

^a^ANCOVA was adjusted for gender, maternal age, parity and video setting

Abbreviations: *PS*: prenatal stress; *Part. Eta Sq.*: partial Eta-squared; *df*: degrees of freedom; *IA*: interaction; *IposMpos*: Infant positive-mother positive; *Ipro-Mpos*: Infant protesting-mother positive; *IproMneg*: Infant protesting-mother negative

When adjusting for additional covariates, the interaction effect of psychosocial PS x episode relating to the IposMpos dyad remained significant (F(1,136) = 4.784, *p* = .030, partial η^2^ = .034). There were no significant effects of the psychosocial PS group on IproMpos and IproMneg (all p’s > .05). When adjusted for additional covariates, the results remained unchanged (see Additional file 1: Table A5).

Psychopathological PS and Perceived PS: No significant main effects were found for either of these stress dimensions (all p’s > .05).

### Impact of HPA axis activity on mother-infant dyadic behavior during the still-face paradigm

#### Cortisol decline

**Fig. 3:**
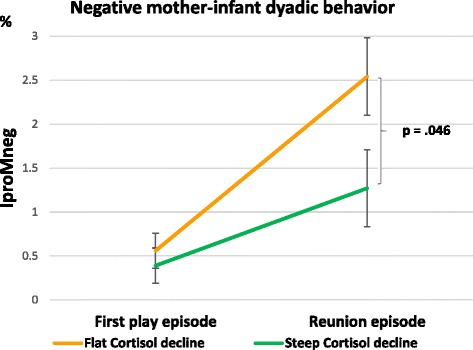
Negative mother-infant dyadic behavior depending on cortisol decline groups in the play and reunion episode (Means and standard errors adjusted for covariates and significant contrasts)

The cortisol decline x episode interaction effect on IproMneg dyadic behavior just reached significance (F(1,126) = 3.949, *p* = .049, partial η^2^ = .030), see Table 3. Moreover, after adjusting for additional covariates, the cortisol decline x episode interaction relating to IproMneg dyadic behavior remained significant (F(1,111) = 4.982, *p* = .028, partial η^2^ = .043). Post-hoc contrasts showed a significant difference between the cortisol decline groups following the still-face manipulation in the reunion episode (*p* = .011) but not in the first play episode (*p* = .163; see Fig. 3). While both mother-infant dyad groups showed an increase in negative dyadic behavior in the reunion episode, the mother-infant dyads whose mothers-to-be had a prenatally flatter decline in cortisol levels exhibited more IproMneg dyadic behavior (M = 2.54; standard error = 4.44) compared to the dyads with a steeper prenatal maternal cortisol decline (M = 1.27; standard error = 0.44). No further significant effects were obtained when adjusting for additional covariates (see Additional file 1: Table A6; all p’s > .05).

**Table 3:** Effect of prenatal HPA axis activity on Infant protesting-mother negative dyadic behavior. Results of ANCOVAs^a^

Effect	IproMneg dyad		
	F/ (df)	p	Part. Eta Sq.
Cortisol decline	3.192 (126)	.076	.025
Episode	0.775 (126)	.380	.006
Cortisol decline x episode IA	3.949 (126)	.049	.029
Cortisol AUCg	3.433 (123)	.066	.027
Episode	0.540 (123)	.464	.004
Cortisol AUCg x episode IA	4.736 (123)	.031	.037

^a^ANCOVAs were adjusted for gender, maternal age, parity and video setting

Abbreviations: *Part. Eta Sq.*: partial Eta-squared; *df*: degrees of freedom; *IA*: interaction; *IposMpos*: Infant positive-mother positive; *IproMpos*: Infant protesting-mother positive; *IproMneg*: Infant protesting-mother negative; *AUCg*: area under the curve with respect to ground

#### Cortisol area under the curve with respect to ground (AUCg)

**Fig. 4:**
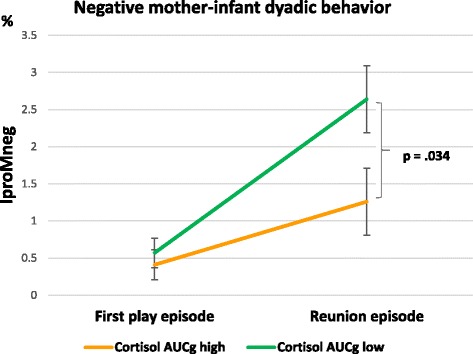
Negative mother-infant dyadic behavior depending on AUCg groups in the play and reunion episode (Means and standard errors adjusted for covariates and significant contrasts)

**Fig. 5:**
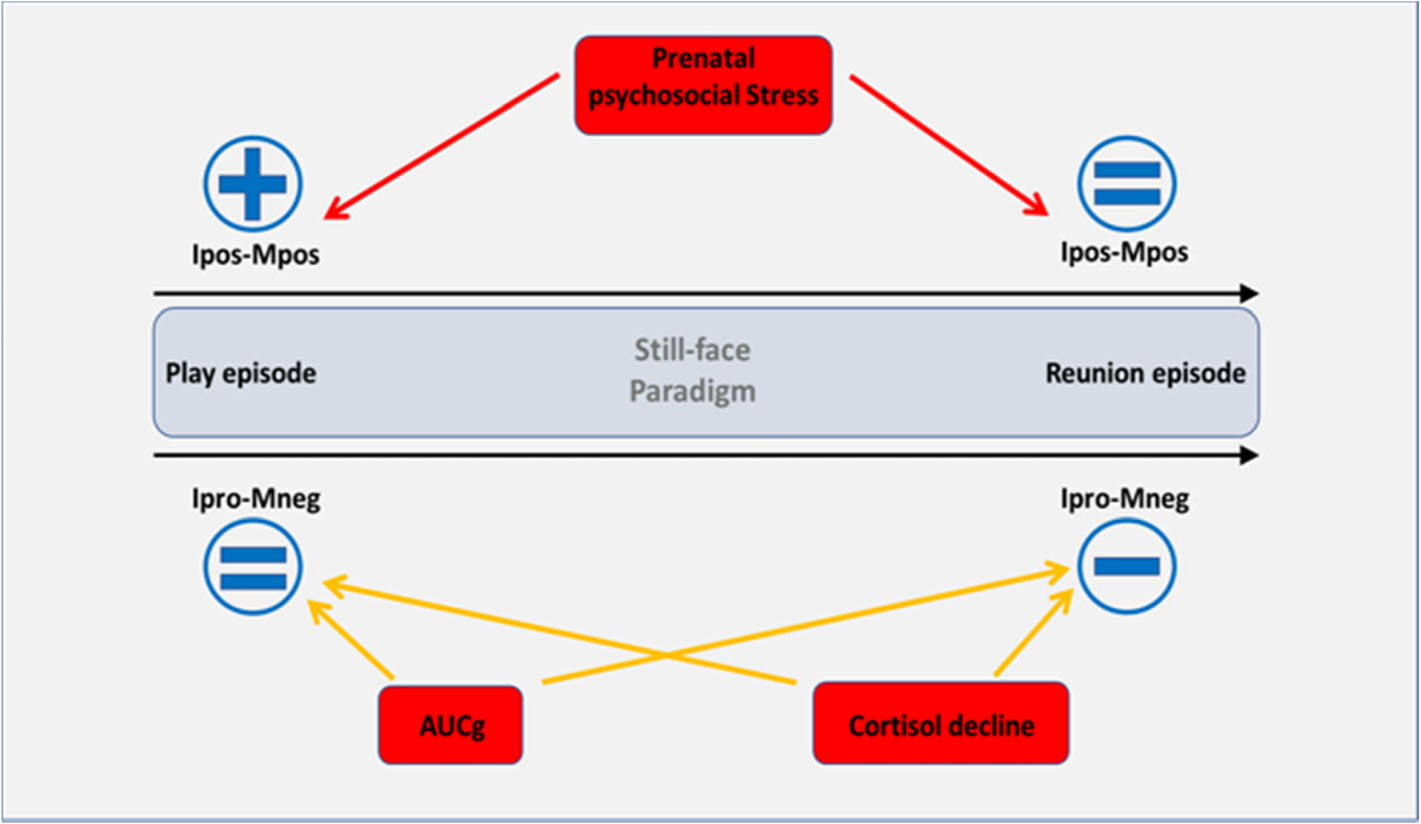
Summary of the present findings. IposMpos: Infant positive-mother positive, IproMneg: Infant protesting-mother negative, AUCg: Area under the curve with respect to ground

An interaction effect of the AUCg x episode relating to the IproMneg dyad emerged (F(1,123) = 4.736, *p* = .031, partial η^2^ = .037); see Fig. 4 and Table 3. When controlling for additional covariates, this effect remained significant (F(1,109) = 5.242, *p* = .024, partial η^2^ = .046). Post-hoc tests showed that there were significant associations between higher diurnal cortisol AUCg levels and the mother-infant dyads in the reunion episode (*p* = .039), but not in the first play episode (*p* = .607). Mother-child dyads with higher maternal diurnal cortisol AUCg levels showed only half as much (M = 1.23; standard error = 0.45) negative dyadic behavior as the less stressed mother-child dyads during the reunion episode (M = 2.64; standard error = 0.45), see Fig. 4. No interaction effects were found of AUCg x episode relating to the IposMpos dyads or the IproMpos dyads (all *p*’s > .05). Finally, when adjusted for all covariates, there were no significant main effects of AUCg on either mother-infant dyad group (all *p*’s > .05). For a summary of the present findings, see Fig. 5.

#### Mediation analyses

Mediation analyses (adjusted for the covariates gender, parity, maternal age and video setting) were computed to examine whether maternal negative behavior mediates the relationship between maternal cortisol decline and negative infant behavior during the reunion episode. The results indicated that cortisol decline was a significant predictor of infant negative behavior during the reunion episode (b = −.023, SE = .011, *p* = .038), but not of maternal negative behavior (b = −.023, SE = .014, *p* = .095). In contrast, maternal negative behavior did not significantly predict infant negative behavior during the reunion episode (b = −.151, SE = .078, *p* = .053). The total effect and the indirect effect were also nonsignificant (*p* > .05). When adjusting for further covariates, cortisol decline remained a significant predictor of infant negative behavior during the reunion episode (b = − .032, SE = .014, *p* = .018), with results showing a significant total effect (b = −.028, *t* = −2.049, *p* = .042) and thus indicating no mediating factors. Analyses computed to examine a potential mediation effect of maternal positive behavior on the relationship between psychosocial stress and infant positive behavior in the reunion episode did not show any significant direct, indirect or total effects (see Additional file 1). Moreover, mediation analyses testing maternal negative behavior as a possible mediator between maternal AUCg and infant negative behavior during the reunion episode failed to show any significant results (see Additional file 1).

## Discussion

The present study used the well-known still-face paradigm within mother-infant interaction to examine infants’ emotion regulation abilities [39]. Evidence emerged for the well-known “still-face effect” and the “carry-over effect” (i.e., increase in negative infant behavior following still-face exposure and consequent decrease in positive dyadic behavior during reunion [38, 39]). Significant effects of the still-face paradigm were shown separately for infant positive and negative behavior, demonstrating the effectiveness of the still-face episode. Regarding mother-infant dyadic behavior, effects were found for both positive and negative interaction patterns and with respect to both psychological and physiological prenatal stress. While mother-infant dyads with high psychosocial PS showed significantly more positive dyadic behavior (i.e. IposMpos) in the first play episode, they did not differ from the low-stress group in the reunion episode. In contrast, the effects of physiological prenatal stress were restricted to negative interaction patterns. Mother-infant dyads with a flatter cortisol decline displayed a more pronounced increase in negative dyadic behavior in the reunion episode compared to those with a steeper prenatal maternal cortisol decline. However, in mother-infant dyads with lower diurnal cortisol AUCg levels, the increase in negative dyadic interaction patterns during reunion was more marked than in those with higher maternal diurnal cortisol AUCg levels. Dyads with low cortisol levels showed about twice as much negative dyadic behavior as the more stressed mother-child dyads during the reunion. Taken together, mothers with a steep HPA decline and high cortisol AUCg in pregnancy showed more positive dyadic interaction patterns following the still-face episode.

The significant effects found in the analyses may suggest an advantageous influence of higher prenatal maternal stress levels, supporting the “stress inoculation” theories, but should be considered in detail. Mother-infant dyads with lower maternal prenatal psychosocial stress showed approximately the same amount of mother-infant positive dyadic behavior (IposMpos) in both play episodes. One explanation for the finding that in contrast to the high-stress group, dyads from the low-stress group did not adjust their positive interaction behavior to the second play episode might be that the decrease in positive dyadic behavior resulted from the “still-face” and the subsequent “carry-over” effect. Several studies have demonstrated a change from infant positive behavior in the first play episode to increased infant negative affect during the still-face episode with less gazing to their mother, as well as the “carry-over” effect in the reunion episode, indicating only a partial decrease of negative infant affect compared to the first play episode [39, 57, 65]. The separate analyses of infant behavior only showed significant episode effects, while no such effects were found when mother-infant dyadic behavior was analyzed. This could be due to the mothers’ consistent amount of positive behavior in the two play episodes: Consistent maternal positive behavior may have merged with the mother-infant dyadic behavior, thus potentially biasing the existing episode effect for infant behavior. Previous research also failed to find significant changes in maternal behavior in the two play episodes [65].

A second reason for these findings might be that mothers with higher psychosocial PS are more likely to try to compensate for the experienced stress by paying more attention to their own behavior, such as displaying more positive attention and behavior towards their child. At the same time, however, these mothers might be more vulnerable to current stressors (i.e., still-face episode), resulting in the reported diminished positive dyadic behavior in the reunion episode. Nevertheless, despite the decrease in positive dyadic mother-infant behavior from the first play to the reunion episode, mother-infant dyads with high psychosocial PS still showed slightly more positive dyadic behavior (M = 3.96; standard error = 0.47) than those with low psychosocial PS (M = 3.75; standard error = 0.47) in the reunion, which puts the significant interaction effect into perspective. When comparing this distinct decrease in positive dyadic behavior in the high psychosocial PS group between the first play episode and the reunion, our results are in line with a previous study [36] reporting that higher dyadic synchrony in the first play was predictive of more negative infant behavior in the reunion. We agree with the potential explanations speculated by these authors, such as that infants with higher synchrony in normal face-to-face interaction with their caregivers might be more distressed when experiencing the loss of synchrony during the still-face episode, resulting in an increase in negative behavior in the reunion [36]. Nevertheless, positive infant behavior (i.e., smiling, laughing) is discussed as a possible regulator of arousal, which is trained on an everyday basis through the interplay in the caregiver-infant dyad, thus enhancing emotion regulation abilities with every positively overcome challenge of dysregulation and short disruption [3, 39].

Regarding prenatal cortisol measures, the findings also suggested a possible beneficial influence of higher prenatal maternal diurnal cortisol area under the curve levels. Mother-infant dyads with lower diurnal cortisol area under the curve levels before birth displayed significantly more negative dyadic behavior during reunion than dyads with higher levels. In contrast, mother-infant dyads with a steeper (“more healthy”) prenatal maternal cortisol decline exhibited less Infant protesting-mother negative dyadic behavior in the reunion than the dyads with a flatter (“less healthy”) decline. It seems that a high amount of HPA axis activity over the course of the day might not be particularly detrimental in the last trimester of pregnancy, as long as there is a decline in the cortisol measures over the day. This is in line with previous research reporting beneficial effects of elevated maternal cortisol in late gestation, resulting in accelerated child development, but not in early pregnancy [66].

Furthermore, dyads with a prenatally steeper cortisol decline did not significantly differ from those with a flatter decline in the first play episode, but did differ in the reunion episode, suggesting that mother-infant dyads with a steep decline are better at handling current stressors (i.e., still-face episode). These findings support the stress inoculation theories. Moreover, they lead to the assumption that mother-infant dyads with higher levels of prenatal maternal cortisol (AUCg) and a steeper diurnal cortisol decline might have an enhanced resilience to current stress or enhanced stress management strategies, both of which were found to be associated with a steeper cortisol decline [67].

Mediation analyses examining possible mediating effects of maternal behavior on the relationship between PS and infant behavior in the reunion episode did not reveal significant effects. Thus, they did not confirm the results of previous research revealing a significant influence of maternal responsive behavior on infant positive behavior in the reunion episode [68], highlighting possible postnatal influencing factors.

The present study indicated significant effects of both stress measures (i.e., psychosocial stress and HPA axis activity). These results correspond well with the “match/(mis-)match hypothesis”, which posits that the offspring benefits from the influence of its early environment if the later environment matches and provides the same demands and resources [32, 33]. Mother-child dyads with higher psychosocial PS and higher prenatal maternal HPA axis activity exhibited less negative dyadic behavior when currently stressed six months after birth compared to dyads with less psychosocial PS and less maternal HPA axis activity, suggesting that the environment matches. Contrary to our hypothesis that changes in the HPA axis would affect maternal and infant behavior, as found in previous research [22, 29], no significant effect of prenatal maternal HPA axis activity on dyadic positive mother-infant behavior was found. This might be due to the “still-face” effect and the general decrease in infant positive and increase in infant negative behavior during the still-face episode. In principle, prenatal maternal cortisol can be associated with both infant behavior [69] and maternal caregiving behavior [70]. However, previous research also reported a lack of associations between self-reported stress and maternal or fetal cortisol levels [71]. Moreover, the timing of prenatal exposure to maternal cortisol seems to have an important influence on its potential beneficial or detrimental impact [66]. Referring to Bolten et al. [69], it has to be conceded that these authors exclusively focused on self-regulation behavior codes of the children, which we did not examine in our study and did not include in the coding of positive and negative dyadic mother-infant behavior.

Furthermore, attenuated cortisol responses were also found to be associated with stress reactivity [72–74]. Recent research on resilience factors has shown that even severe early life stress was not necessarily linked to a hyper-responsive stress and fear system [75], although severe adverse early life experiences are still seen as a contributor to adult psychopathology [76]. Moreover, the postnatal environment can moderate the relationship between PS and later behavioral outcome, being able to both worsen and reverse the influence of ELS [77].

Finally, individual differences need to be taken into account. Research in rodents demonstrated both beneficial and impairing effects of prenatal stress depending on the strain of rats [78] or the amount of stress experienced [79]. Concerning the dosage of stress, DiPietro [80] argued that the resulting impact of prenatal stress on infants’ development could be akin to the relation between arousal and performance reflected in the U-shaped function of the “Yerkes-Dodson law”, with a moderate dosage being seen as optimal.

Keeping in mind that the results presented above only showed a snapshot of mother-infant behavior at six months postpartum, further research is needed to identify individual factors and general changes in the impact of PS during infant development. Despite the reports of potentially beneficial influences of prenatal stress exposure, the majority of findings suggesting an impairing influence of early life stress should not be neglected. Furthermore, research on “allostatic load” has suggested that former resilience can turn into proneness to later diseases [81]. Following the assumption, better survival in stressful and dangerous environments might come at the cost of a shorter lifespan and vulnerability to disorders and diseases later on [82].

Several limitations of the present study need to be taken into account. First, the cortisol data were collected and self-reported by the mothers. For this reason, we set up a strict limitation of outliers. The cortisol measures seem to lie in a normal range expected for mothers-to-be in the third trimester of pregnancy [83], possibly less influenced by the reported maternal stress than by the pregnancy itself. Second, the dichotomized stress measurement (extreme-) groups showed an amount of overlapping data for the mother-infant dyadic behavior, defined by means and standard deviations, which has to be taken into account. Third, the study consisted of healthy non-inpatient women. Therefore, it would not be appropriate to compare findings from our sample of pregnant women exposed to rather moderate prenatal maternal stress levels with studies investigating severe event-related prenatal stress in mothers-to-be (i.e.; catastrophes, current psychological disorders needing inpatient treatment). Fourth, prenatal stress can be mediated by influencing factors such as maternal sensitivity, infants’ temperament, coping abilities or attachment quality [84, 85], none of which were controlled for in the current study. Finally, as our study is the first to attempt to elucidate influences of prenatal stress on mother-infant dyadic behavior, it is therefore of a hypothesis-generating and exploratory nature. Hence, *p*-values were not post-hoc corrected for multiple testing and the reported results need to be replicated and verified in further independent controlled experiments.

## Conclusion

Mother-infant dyads exposed to higher levels of prenatal psychosocial stress showed more positive dyadic behavior during the play episode, while mother-infant dyads with higher diurnal cortisol and a steeper cortisol decline displayed less negative dyadic behavior during the reunion episode than the respective comparison groups. Overall, these results support the “*stress inoculation*” theories, which report beneficial effects of prenatal stress [28, 30, 86] as well as the *“match/(mis-) match hypothesis*” [32, 33], contributing to the exploration of resilience and emotion regulation abilities. Nevertheless, with the vast amount of studies reporting impairing influences of prenatal stress, findings of possible positive influences should be taken into consideration but treated with caution and subject to verification. The mixed research findings examining the impact of prenatal stress on infants` development require further research to elucidate the reasons for the conflicting findings.

## Additional file

Additional file 1: Inclusion and exclusion criteria of video data. Table A1: Means, standard deviations and range of the ICEP codes. Assessment of the stress indices. Table A2: Pearson correlations between the mother-infant dyadic behavior categories across the still-face episodes (*N* = 164). Table A3: Pearson correlations between the prenatal psychological stress indices and the postnatal Perceived Stress Scale (PSS) (*N* = 164). Table A4: Pearson correlations between the psychosocial stress and psychophysiological stress indices (*N* = 134). Table A5: Effect of psychosocial PS on mother-infant dyadic behavior. Results of ANCOVAs additionally adjusted for breastfeeding, maternal depression before birth (except for psychopathological ELS), Apgar score after 5′, perinatal complications and current perceived stress in high-stress and low-stress groups. Table A6: Effect of maternal HPA axis activity on mother-infant dyadic behavior. Results of ANCOVAs additionally adjusted for breastfeeding, maternal depression before birth, Apgar score after 5′, perinatal complications and current perceived stress in high-stress and low-stress groups. Results of mediation analyses. (DOCX 36 kb)

## Abbreviations

11ß-HSD2: 11ß-hydroxysteroid dehydrogenase 2
ACTH: adrenocorticotropic hormone
ANCOVA: analyses of covariance
AUCg: area under the curve with respect to ground
BPD: Borderline personality disorder
df: Degrees of freedom
DNA: Deoxyribonucleic acid
FFE: Face-to-face play episode/ first play episode
FI: Saliva collection immediately after awakening
FII: Saliva collection 30 min after getting up
FIII: Saliva collection 14 h after awakening
HIV: human immunodeficiency virus
HPA axis: hypothalamic-pituitary-adrenocortical axis
HPA: Hypothalamic-pituitary-adrenal axis
IA: interaction
ICEP: Infant and Caregiver Engagement Phases
IposMpos: Infant positive-mother positive dyad
IproMneg: Infant protesting-mother negative dyad
IproMpos: Infant protesting-mother positive dyad
M: mean
Part. Eta sq.: Partial Eta-squared
POSEIDON: Pre-, Peri- and POstnatal Stress: Epigenetic Impact on DepressiON
PS: Prenatal stress
PSS: Perceived Stress Scale
RE: Reunion episode
SD: Standard deviation
