# Supplementary material for: Impact of prenatal stress on mother-infant dyadic behavior during the still-face paradigm
Source: Borderline Personal Disord Emot Dysregul. 2018 Jan 22;5:2. doi: 10.1186/s40479-018-0078-8 (PMC5778796; doi:10.1186/s40479-018-0078-8)
Supplement: Additional file 1: — Inclusion and exclusion criteria of video data. Table A1: Means, standard deviations and range of the ICEP codes. Assessment of the stress indices. Table A2: Pearson correlations between the mother-infant dyadic behavior categories across the still-face episodes (N = 164). Table A3: Pearson correlations between the prenatal psychological stress indices and the postnatal Perceived Stress Scale (PSS) (N = 164). Table A4: Pearson correlations between the psychosocial stress and psychophysiological stress indices (N = 134). Table A5: Effect of psychosocial PS on mother-infant dyadic behavior. Results of ANCOVAs additionally adjusted for breastfeeding, maternal depression before birth (except for psychopathological ELS), Apgar score after 5′, perinatal complications and current perceived stress in high-stress and low-stress groups. Table A6: Effect of maternal HPA axis activity on mother-infant dyadic behavior. Results of ANCOVAs additionally adjusted for breastfeeding, maternal depression before birth, Apgar score after 5′, perinatal complications and current perceived stress in high-stress and low-stress groups. Results of mediation analyses. (DOCX 36 kb) [file 40479_2018_78_MOESM1_ESM.docx]

**Additional files**

**Impact of prenatal stress on mother-infant dyadic behavior during the still-face paradigm.**

**Additional File 1: Inclusion and exclusion criteria of video data**

Several video sets had to be excluded due to technical problems of the filmed material (i.e., light and sound overexposure, missing sequences, early ending; n = 8) and outliers in infant behavior assumed as disturbed due to interfering circumstances (i.e., sleepiness and >80% infant protesting behavior; n = 28; cf. baseline of 10% negative infant behavior reported by [1]), leaving 164 mother-infant pairs for statistical data analysis.

**Additional File 2: Table A1**

Table A1: **Means, standard deviations and range of the ICEP behavior codes**

|  | **Play episode** | | **Reunion Episode** | |
| --- | --- | --- | --- | --- |
| **ICEP Behavior Codes (n = 164)** | **M (SD)** | **Range** | **M (SD)** | **Range** |
| Infant positive (Ipos) | 18.54 (15.53) | Min: 0  Max: 60.81 | 15.15 (14.54) | Min: 0  Max: 63.95 |
| Infant protest (Ipro) | 5.47 (12.17) | Min: 0  Max: 49.78 | 23.17 (31.59) | Min: 0  Max: 100 |
| Mother positive (Cpos and Cpvc) | 77.25 (18.04) | Min: 7.07  Max: 100 | 70.79 (20.21) | Min: 5.91  Max: 100 |
| Mother negative (Cint, Cneu, Cnon) | 21.75 (17.29) | Min: 0  Max: 92.91 | 27.32 (19.43) | Min: 0  Max: 94.09 |
| intrusive behavior (Cint) | 13.23 (15.09) | Min: 0  Max: 89.93 | 15.14 (17.44) | Min: 0  Max: 85.48 |
| social monitor/neutral vocalization (Cneu) | 8.35 (9.99) | Min: 0  Max: 51.58 | 11.62 (11.82) | Min: 0  Max: 52.39 |
| non-infant-focused engagement (Cnon) | .17 (.53) | Min: 0  Max: 3.42 | .56 (.431) | Min: 0  Max: 52.39 |

All means and standard deviations are percentages. Abbreviations: ICEP: Infant and Caregiver Engagement Phases

**Additional File 3: Assessment of the stress indices**

Mothers-to-be (N = 410) were assessed in the last trimester of pregnancy using a structured interview and a series of questionnaires in order to collect information concerning a broad range of environmental and sociodemographic risk factors, prenatal medical risk factors, general medical characteristics, and psychosocial risk factors. Eight main stressor variables derived from eight different questionnaires were selected to represent a variety of prenatal adversities, yielding three different dimensions of stress: a) maternal psychopathology (primarily depressive and anxiety symptoms); b) perceived stress; and c) socioeconomic and psychosocial stress. The composite score of psychopathology was derived from three questionnaires (Edinburgh Postnatal Depression Scale (EPDS) [2]; State-Trait Anxiety Inventory (STAIT/S) [3]; Anxiety Screening Questionnaire (ASQ) [4], and the Mini International Neuropsychiatric Interview (MINI) [5], indicating current depression or anxiety disorder. The composite score of perceived stress was derived from the Perceived Stress Scale (PSS) [6] and the Prenatal Distress Questionnaire (PDQ) [7]. The composite measure of socioeconomic and psychosocial stress was obtained from the Life Experiences Survey (LES) [8], which scores for negative life events, and the inverted score of the Social Support Questionnaire (Soz-U) [9]. Furthermore, the interview scores for the categories living without a partner, encouragement through partner, separation(s) in the last year, daily arguments, physical conflicts within the preceding 12 months, the composition of the household (e.g. rooms per person), no academic qualification, no professional education, monthly income per household less than 1,750 Euro, and debt were included in the psychosocial stress axis (positively-impacting data were inverted; for detailed records, see Dukal et al. [10]). In addition, an “adversity score” was calculated by summing up the number of dichotomous stressful prenatal adverse conditions and environmental circumstances. To obtain a homogeneous composite measure of prenatal stress, a principal components analysis (PCA) was performed. This involved the eight main stressor variables and the total adversity score as a ninth main variable. This analysis yielded a first principal component (PC1), which explained about 60% of the common variance.

**Additional File 4: Table A2**

Table A2: **Pearson correlations between the mother-infant dyadic behavior categories across the still-face episodes (N = 164).**

|  | | **IposMpos** | **IproMpos** | | **IproMneg** | |
| --- | --- | --- | --- | --- | --- | --- |
|  | | **RE** | **FFE** | **RE** | **FFE** | **RE** |
| **IposMpos** | **FFE** | 0.430; p < .001 | - 0.260; p = .001 | - 0.211; p = .007 | - 0.168; p = .032 | - 0.003; p = .968 |
|  | **RE** |  | - 0.297; p < 001 | - 0.357; p < .001 | - 0.220; p = .005 | - 0.268; p = .001 |
|  |  |  |  |  |  |  |
| **IproMpos** | **FFE** |  |  | | 0.482; p < .001 | - 0.320; p < .001 |
|  | **RE** |  |  |  | 0.282; p < .001 | 0.427; p < .001 |

Abbreviations: FFE: first play / Face-to-face play episode; RE: Reunion episode; IposMpos: Infant positive-mother positive, IproMpos: Infant protesting-mother positive; IproMneg: Infant protesting–mother negative.

**Additional File 5: Table A3**

Table A2. **Pearson correlations between the prenatal psychological stress indices and the postnatal Perceived Stress Scale (N = 164).**

|  | **Psychopathological stress** | **Perceived stress** | **Psychosocial stress** |
| --- | --- | --- | --- |
| **Perceived stress** | .739; p < .001 | 1 |  |
| **Psychosocial stress** | .614; p < .001 | .604; p < .001 | 1 |
| **PSS** | .609; p < .001 | .693; p < .001 | .473; p < .001 |

Abbreviations: PSS: Perceived Stress Scale, surveyed six months after birth.

**Additional File 6: Table A4**

Table A4. **Pearson correlations between the psychosocial stress and psychophysiological stress indices (N = 134).**

|  | **Perceived stress** | **Psychosocial stress** | **Cortisol Decline** | **Cortisol AUCg** |
| --- | --- | --- | --- | --- |
| **Psychopathological stress** | .721;  p < .001;  N = 134 | .629;  p < .001;  N = 134 | - .203;  p = .019;  N = 127 | - .061;  p = .491;  N = 131 |
| **Perceived stress** | 1  N = 134 | .582;  p < .001;  N = 134 | - .003;  p = .974;  N = 127 | .081;  p = .360;  N = 131 |
| **Psychosocial stress** |  | 1  N = 134 | - .184;  p = .033;  N = 127 | - .013;  p = .879;  N = 131 |
| **Cortisol Decline** |  |  | 1  N = 127 | .467;  p < .001;  N = 126 |

Abbreviations: AUCg: area under the curve with respect to ground.

**Additional File 7: Table A5**

Table A5. **Effect of psychosocial PS on mother-infant dyadic positive behavior. Results of ANCOVA additionally adjusted for breastfeeding, maternal depression before birth (except for psychopathological ELS), Apgar score after 5’, perinatal complications, and current perceived stress in high-stress and low-stress groups.**

| **Effect** | **IposMpos dyad** | | |
| --- | --- | --- | --- |
|  | F/ (df) | P | Part. Eta Sq. |
| **Psychosocial PS** | 3.714 (136) | .056 | .027 |
| **Episode** | 0.187 (136) | .666 | .001 |
| **Psychosocial PS x episode IA** | 4.784 (136) | .030 | .034 |

Abbreviations: PS: prenatal stress; Part. Eta Sq.: partial Eta-squared; IA: interaction; Infant positive-mother positive: IposMpos; Infant protesting-mother positive: IproMpos; Infant protesting-mother negative: IproMneg; FFE: Face-to-face episode / first play episode; RE. Reunion episode.

**Additional File 8: Table A6**

Table A6. **Effect of maternal HPA axis activity on infant protesting-mother negative dyadic behavior. Results of ANCOVAs additionally adjusted for breastfeeding, maternal depression before birth, Apgar score after 5’, perinatal complications, and current perceived stress in high HPA axis activity and low HPA axis activity groups.**

| **Effect** | **IproMneg dyad** | | |
| --- | --- | --- | --- |
|  | F/ (df) | P | Part. Eta Sq. |
| **Cortisol decline** | 7.157 (111) | .009 | .061 |
| **Episode** | 1.364 (111) | .245 | .012 |
| **Cortisol decline x episode IA** | 4.982 (111) | .028 | .043 |
|  |  |  |  |
| **Cortisol AUCg** | 5.285(109) | .023 | .046 |
| **Episode** | .565 (109) | .454 | .005 |
| **Cortisol AUCg x episode IA** | 5.242 (109) | .024 | .046 |

Abbreviations: Part. Eta Sq.: partial Eta-squared; IA: interaction; IposMpos: Infant positive-mother positive; IproMpos: Infant protesting-mother positive; IproMneg: Infant protesting-mother negative; FFE: Face-to-face episode / first play episode; RE. Reunion episode; AUCg: area under the curve with respect to ground.

**Additional File 9: Results of mediation analyses**

Mediation analysis (considering the covariates gender, parity, maternal age and video setting) investigating the possible mediating role of positive maternal behavior between PS and positive infant behavior in the reunion play indicated that psychosocial PS was not a significant predictor of infant positive behavior (b = .007, SE = .091, p = .940) or of maternal positive behavior during reunion (b = -.023; SE = .061, p = .710). Furthermore, maternal positive behavior did not significantly predict infant positive behavior in the reunion episode (b = -.137, SE = .091, p = .137). Tests for direct and indirect effect as well as total effects were nonsignificant (all p > .05), suggesting that neither mediator effects nor a predictor-outcome relationship can be assumed. The same applied for the mediation analysis considering further covariates (i.e., breastfeeding, perinatal complications, perceived current stress (PSS), Apgar score five minutes after birth, and maternal depression).

Mediation analyses testing the role of negative maternal behavior in the relation between maternal cortisol AUCg and infant negative behavior indicated that AUCg was not a significant predictor of infant negative behavior (b < .001, SE < .001, p = .138) or of maternal negative behavior (b < .001, SE < .001, p = .197). Further, maternal negative behavior was not a significant predictor of infant negative behavior in the reunion (b = -.126, SE = .075, p =.095). Moreover, tests for total and indirect effects indicated no significance (all p > .05). The same applied for the mediation analysis considering all covariates (all p > .05).

**References**

1. Moore GA, Calkins SD: **Infants' vagal regulation in the still-face paradigm is related to dyadic coordination of mother-infant interaction.** *Dev Psychol* 2004, **40:**1068-1080.

2. Cox JL, Holden JM, Sagovsky R: **Detection of postnatal depression. Development of the 10-item Edinburgh Postnatal Depression Scale.** *Br J Psychiatry* 1987, **150:**782-786.

3. Spielberger CD, Gorsuch RL, Lushene RE: *Manual for the state-trait anxiety inventory.* Palo Alto: Consulting Psychologists Press; 1970.

4. Wittchen HU, Boyer P: **Screening for anxiety disorders. Sensitivity and specificity of the Anxiety Screening Questionnaire (ASQ-15).** *Br J Psychiatry Suppl* 1998**:**10-17.

5. Sheehan DV, Lecrubier Y, Sheehan KH, Amorim P, Janavs J, Weiller E, Hergueta T, Baker R, Dunbar GC: **The Mini-International Neuropsychiatric Interview (M.I.N.I.): the development and validation of a structured diagnostic psychiatric interview for DSM-IV and ICD-10.** *J Clin Psychiatry* 1998, **59 Suppl 20:**22-33;quiz 34-57.

6. Cohen S, Kamarck T, Mermelstein R: **A global measure of perceived stress.** *J Health Soc Behav* 1983, **24:**385-396.

7. Yali AM, Lobel M: **Coping and distress in pregnancy: an investigation of medically high risk women.** *J Psychosom Obstet Gynaecol* 1999, **20:**39-52.

8. Sarason IG, Johnson JH, Siegel JM: **Assessing the impact of life changes: development of the Life Experiences Survey.** *J Consult Clin Psychol* 1978, **46:**932-946.

9. Fydrich T, Sommer G, Brähler E: *Fragebogen zur sozialen Unterstützung : F-SozU.* Göttingen ; Bern ; Wien [u.a.]: Hogrefe; 2007.

10. Dukal H, Frank J, Lang M, Treutlein J, Gilles M, Wolf IA, Krumm B, Massart R, Szyf M, Laucht M, et al: **New-born females show higher stress- and genotype-independent methylation of SLC6A4 than males.** *Borderline Personal Disord Emot Dysregul* 2015, **2:**8.
